# Microfilariae infection by *Acanthocheilonema reconditum* and *Dirofilaria immitis* and their molecular detection in a dog with lymphoma: Case report

**DOI:** 10.5455/javar.2023.j701

**Published:** 2023-09-30

**Authors:** Rodrigo Lugo-Vargas, Ruben Dario Perez-Ramirez, Nicolas Carrillo-Godoy, Iang Schroniltgen Rondón-Barragán

**Affiliations:** Laboratory of Immunology and Molecular Biology, University of Tolima, Ibagué, Colombia

**Keywords:** 5.8s-ITS2-28s, *Acanthocheilonema reconditum*, *COX1*, *Dirofilaria immitis*, lymphoma

## Abstract

**Objective:**

Microfilariae parasites are common in tropical regions, and some species are reported as potentially zoonotic. The diagnosis of filarial infection in dogs by cytology or hematologic techniques showed lower sensibility and specificity, which may result in misdiagnosis. Thus, molecular techniques seem to be an alternative to identifying and detecting microfilariae infections. On the other hand, lymphoma is one of the main tumors in domestic animals, with a high prevalence in domestic canines. This study aims to report a mixed infection with microfilariae in a dog with lymphoma, emphasizing its diagnosis and the possible role of this infection in the development of the neoplasia.

**Materials and Methods:**

An 8-year-old male mixed breed dog was referred to consultation due to the presence of lethargy, recumbency, skin ulceration lesions, nonspecific pain manifestations, emesis, myoclonus in the left temporalis muscle, and seizures. Routine blood and biochemistry tests were normal, and cytology of the skin evidenced a microfilariae infection. The dog died due to a cardiorespiratory arrest, and tissue sampling was done for histopathology and molecular analysis at the necropsy examination.

**Results:**

Skin lesions were related to a microfilarial pyogranuloma related to *Acanthocheilonema reconditum*. Histopathology of the spleen and liver revealed a diffuse lymphoma composed of blast cells and large lymphocytes, distributed diffusely in the parenchyma and surrounding the vasculature. In the skin, microfilariae were seen in some superficial capillaries.

**Conclusion:**

This study describes a microfilariae mixed infection with *A. reconditum *and* Dirofilaria immitis* in a dog with a lymphoma and its molecular detection. To the knowledge of the authors, this is the first report of a mixed microfilariae infection in a tumor of a dog and highlights the use of molecular techniques, i.e., polymerase chain reaction, for an accurate diagnosis.

## Introduction

Filarial worms can infect canines as the definitive host; they belong to the order Spirurida*,* superfamily Filarioidea*,* and the hematophagous arthropods can be vectors and intermediate hosts. Several species can be found, including *Dirofilaria immitis,* *Dirofilaria repens* (Dirofilariinae subfamily), *Acanthocheilonema reconditum*, *Amphiachyris dracunculoides,* and *Cercopithifilaria* spp., (Onchocercinae subfamily) [1]. Discrimination of microfilarial species is performed through concentration techniques such as morphometry, histochemical assays, and molecular techniques. Still, it can be challenging, mainly in areas where different filarioids are present in sympatry [2–4]. In Colombia, *D. immitis* has been detected by immunochromatography and ELISA [5]. However, reports focused on the molecular diagnosis of *A. reconditum* are scarce [6].

On the other hand, lymphoma (malignant lymphoma or lymphosarcoma) is one of the most common tumors in dogs, with an annual incidence estimated to range between 13 and 114 per 100,000 dogs at risk [7]. The multicentric form is the most common clinical presentation, which affects the peripheral lymph nodes; however, extranodal forms such as abdominal, ocular, cutaneous, mediastinal, central nervous system, and pulmonary lymphoma [8].

In human patients, the coexistence of neoplasia and filarial organisms has been described, mainly in endemic areas, but the cause is under discussion [9–11]. The cytological and histopathological analyses have revealed filariae inside the primary tumor area and in the metastasis. In these cases, the patients are asymptomatic, rarely showing eosinophilia associated with the presence of the parasite [11]. In this study, a case of infection with *D. immitis* and *A. reconditum* in a dog with lymphoma is described along with its diagnosis by cytological, histopathological, and molecular methods.

## Case Presentation

An 8-year-old male mixed breed dog was referred to consultation by his owner due to lethargy remaining in recumbency, skin ulceration lesions, nonspecific pain manifestations, emesis, and myoclonus in the left temporalis muscle. Fifteen days before the consultation, the dog was treated with phenobarbital and B complex due to the presence of seizures, which persisted even after treatment. Since the patient previously presented coughing episodes, the presence of hemoparasites was considered.

### Clinical examination

Upon physical examination, physiological constants were within reference values, and the patient ate and drank water normally. In addition, complete hemogram analysis, serum biochemistry, skin cytology, and a canine distemper virus (CDV) antigen rapid test (Vcheck CDV ag-Bionote, Mexico) were performed. For skin cytology, imprints were made from the ulcerated areas by lifting the scar and imprinting it with the slide, allowing it to dye and stain with Wright (IHR Ltd., Colombia).

Due to the suspicion of hemoparasites, a Woo test and a blood smear that was stained with Wright were performed. First, canine whole blood was transferred to capillaries without heparin, and then the end of the tube was heat-sealed and centrifuged at 13,700×*g* for 5 min. Morphological characterization was performed in a binocular microscope (MRP-3000, Scientific USA) at 5× and 10× magnification for the Woo test and 100× for the smear.

The patient did not show any alteration in the hemogram, and it was negative for the CDV test; however, due to its symptoms, it was decided to keep the patient under observation and start a pain treatment with multimodal analgesia (fentanyl, ketamine, lidocaine, and dipyrone) at night. The next morning, the patient showed no improvement; thereby, tramadol was administered. Despite the treatment, the patient died due to cardiopulmonary arrest. A necropsy examination was carried out, and samples from the liver, spleen, kidney, urine, brain, cerebellum, cerebrospinal fluid, and skin were collected for histopathology and molecular diagnosis. All the procedures, including sampling collection, followed the guidelines for research ethics and animal welfare based on resolution number 8,430/1,993 and law 84/1,989 and fulfilled the guidelines for animal care and use in clinical research and teaching.

To carry out the molecular diagnosis of hemoparasites, genomic DNA (gDNA) was extracted from tissue samples using the E.Z.N.A.^®^ Tissue DNA Kit (Omega Bio-tek, USA) following the manufacturer’s instructions. The quality of the extracted DNA was verified by spectrophotometry (NanoDrop One, ThermoFisher, USA) and amplification of canine *beta actin* (*ACTB*). Detection of microfilariae was performed by amplifying ribosomal RNA (rRNA) 5.8s-ITS2-28s, using primers flanking conserved areas of the gene. To discriminate between filarial species, the cytochrome oxidase C subunit 1 (*COX1*) gene from *D. immitis* and *A. reconditum* was amplified (Table 1). To identify *Wolbachia* spp., an endosymbiont bacterium, primers targeting the *Wolbachia *surface protein (*wsp*) gene were used (Table 1).

**Table 1.** Primers sequences for amplification of microfilarial and *Wolbachia* spp. genes.

Gen/Organism	Primers (5¢—3¢)	Ta* (°C)	Amplicon size (pb)	Reference
*ACTB/* *Canis lupus familiaris*	F-GGCTACAGCTTCACCACCAC	60.9	497	This study
	R-TACTCCTGCTTGCTGATCCACA	61.4		
*COX1/* *A. reconditum*	F:ATCTTTGTTTATGGTGTATC	50	589	[6]
	R:ATAAGTACGAGTATCAATATC			
*COX1/* *D. immitis*	F:ACCGGTGTTTGGGATTGTTA	50	169	
	R:ATAAGTACGAGTATCAATATC			
*5.8s-ITS2-28s *rRNA*/* *A. reconditum* *Dirofilaria immitis* *Dirofilaria repens*	F:AGTGCGAATTGCAGACGCATTGAG	58	577 542 484	
	R:AGCGGGTAATCACGACTGAGTTGA			
*wsp/* *Wolbachia* spp.	F:TGGTCCAATAAGTGATGAAGAAACTAGCTA	50	595	
	R:AAAATTAAACGCTACTCCAGCTTCTGCAC			

* Ta: Annealing temperature.

Polymerase chain reaction (PCR) was carried out using a reaction volume of 25 μl, composed of 5× standard reaction buffer (5 μl), 1.5 mM dNTPs (2 μl), primers forward and reverse (1 μl of each one, 10 pmol/μl), 0.15 μl of OneTaq^®^ DNA Polymerase (New England BioLabs, Madison, WI), gDNA sample (1 μl) and distilled and deionized water (14.85 μl). Amplification was performed in a thermocycler (ProFlex PCR System, Applied Biosystems, USA) with a first denaturation cycle for 3 min at 94°C, followed by 35 cycles of denaturation at 94°C for 30 sec, annealing at specific temperatures (Table 1) for 30 sec, extension for 30 sec at 68°C for all reactions, except for the amplification of the *5.8s-ITS2-28s* rRNA fragment, in which 1 min was set, and 1 final extension cycle at 68°C for 5 min.

PCR amplicons were revealed by horizontal agarose gel electrophoresis (2%), stained with Hydragreen (ACTGene, USA), at 100 volts for 40 min using the MyGel Mini electrophoresis chamber (ACCURIS, USA), and visualized through the ENDURO GDS™ gel documentation system (LabNet Intl, USA) under ultraviolet light.

**Figure 1.**
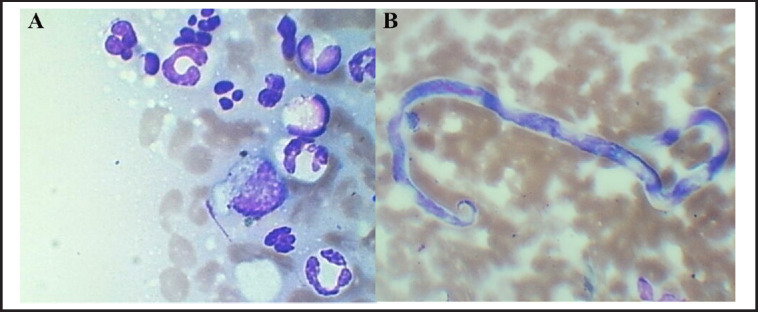
Cytology of skin smear. (A) Hemorrhagic background with abundant neutrophils, presence of macrophages and lymphocytes. (B) Microfilariae specimen with blunt head and hook-shaped tail.

In the cytology of ulcerative lesions of the skin of the right hindlimb, a hemorrhagic background with neutrophil predominance, the presence of macrophages, and the absence of bacteria were noted (Fig. 1a). Likewise, filarioid structures characterized by a blunt head and hook-shaped tail were observed (Fig. 1b). Concluding that the skin lesions were related to a microfilarial pyogranuloma related to *A. reconditum. *The morphological characteristics of the parasites observed in the Woo test were similar, including a cephalic region and a filiform ending. In addition, progressive and stationary movements were observed.

**Figure 2.**
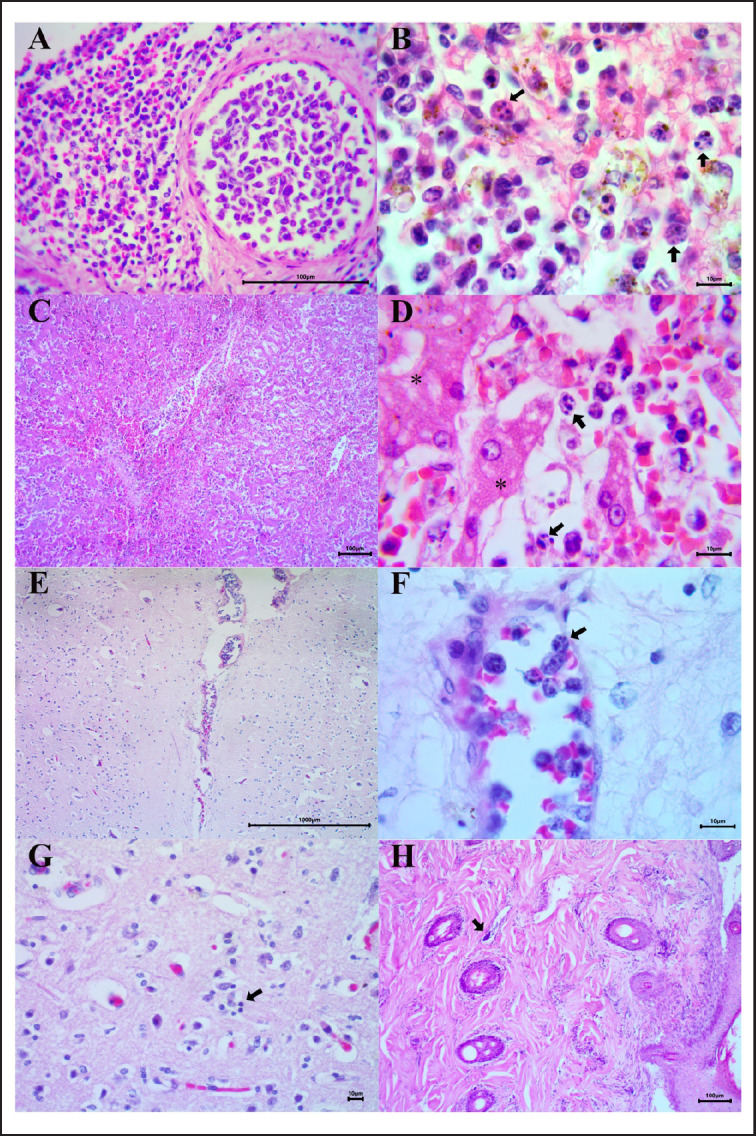
Histopathological findings of the patient. (A) Spleen, diffuse lymphocytic neoplasia, with an abundance of blast lymphocytic cells and large hyperchromatic lymphocytes. (B) Spleen, lymphocytes with lobated nuclei, irregular chromatin, and numerous large nucleoli (arrows). (C) Liver, centrilobular congestion with sinusoids filled with large hyperchromatic lymphocytes. (D) Liver, hepatocytes showed hyaline vacuolization (asterisks), sinusoidal space is filled with lymphocytes with numerous large nucleoli (arrows). (E) Brain, lumen of leptomeningeal and intraparenchymal venules and veins with abundant infiltration of large lymphocytic cells. (F) Brain, large lymphocytes (arrow) associated to endothelium. (G) Brain, slight spongiosis of the neuropil, neuronal peripheral chromatolysis, and satellitosis (arrow). (H) Skin, fibrosis with a mild multifocal leukocyte infiltrate. Microfilariae can be seen in the superficial capillaries (arrow).

Histopathological analysis of the spleen revealed a diffuse lymphoma composed of round cells, blast cells, and large lymphocytes, distributed diffusely in the parenchyma and surrounding the vasculature, megakaryocytes, hemosiderophages, and plasma cells were also evidenced (Fig. 2a). The neoplastic cells showed slightly eosinophilic cytoplasm, ovoid and vesicular nuclei with hyperchromatism, marginal nucleoli, and frequent mitotic figures (Fig. 2b). In the liver and the brain, vascular congestion with the presence of large mononuclear round cells similar to those described in the spleen was detected (Fig. 2c and d). In addition, brain tissue showed mild spongiosis of the neuropil and mild peripheral chromatolysis (Fig. 2e–g).

Skin showed marked dermal interstitial and perianexal fibrosis and a mild multifocal leukocyte infiltration with the presence of lymphocytes, plasma cells, and scarce neutrophils distributed in some pilosebaceous and epitrichial units and the superficial capillaries (Fig. 2h). Microfilariae are seen in some superficial capillaries. Finally, an epidermis with mild orthokeratotic hyperkeratosis was observed*.*

**Figure 3.**
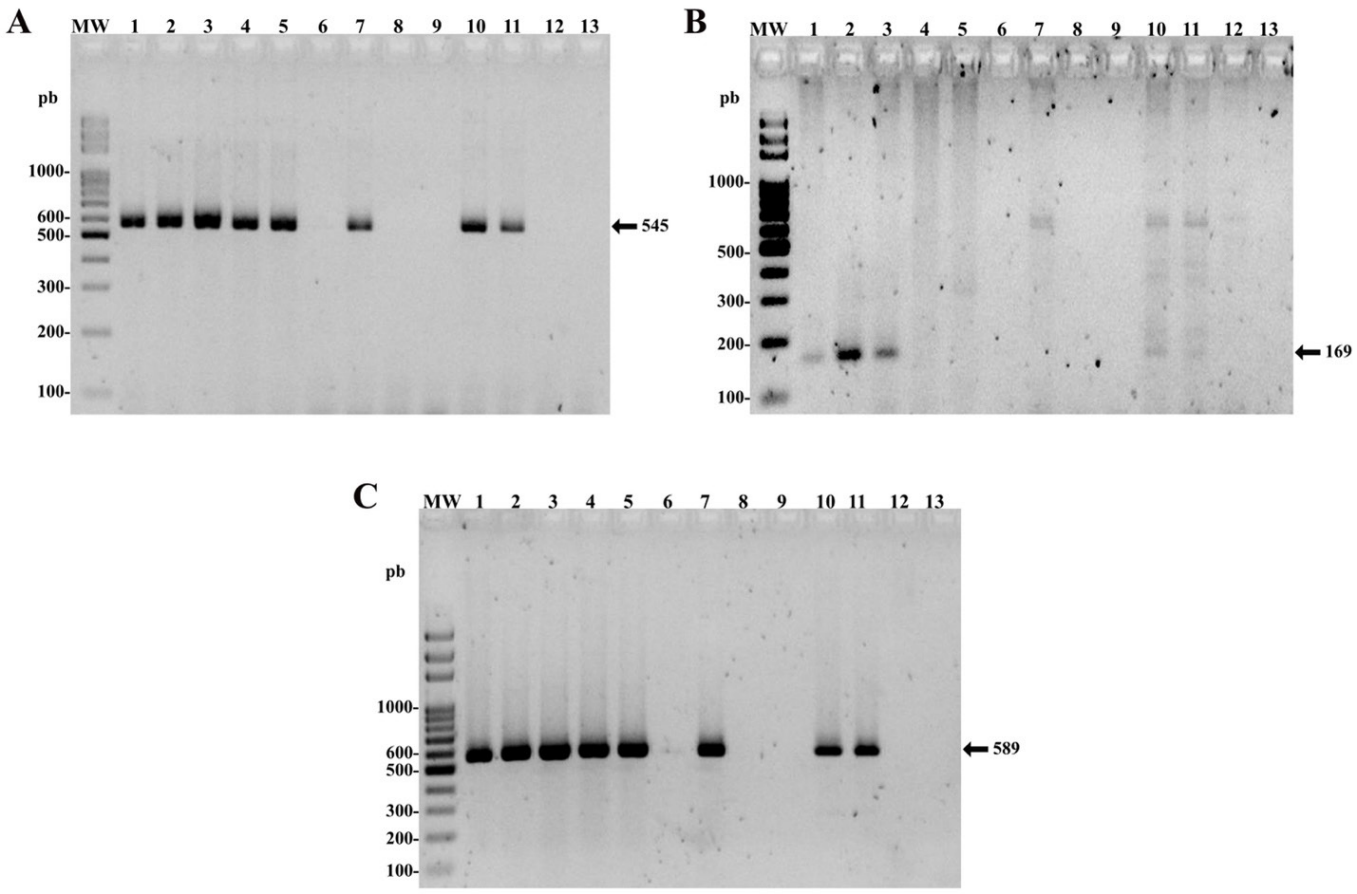
Detection of microfilariae by PCR in several tissues of the dog patient. (A) panfilarial* 5.8s-ITS2-28s *rRNA gene fragment (545 pb). (B)* Acanthocheilonema reconditum COX1* gene fragment (589 pb). (C) *Dirofilaria immitis COX1* gene fragment (169 pb). Tissue samples are organized as follows: skin (1), lung (2), liver (3), kidney (4), spleen (5), brain (6), cerebellum (7), urine (8), cerebrospinal fluid (9), blood sample 1 (10), blood sample 2 (11), negative control, negative patient (12) and no template negative control (13). Molecular weight marker (MW) (Solis Biodyne, Slovenia). Agarose gel (2%).

*ACTB* gene was amplified from all samples (data not shown) with a size of approximately 497 bp, indicating adequate gDNA quality. *5.8s-ITS2-28s *rRNA was amplified to determine the possible species of microfilariae in the canine patient, with similar amplicon sizes for *D. immitis* and *A. reconditum* (Fig. 3a). By amplifying the *COX1* fragments corresponding to each filarial species, it was shown that the kidney, spleen, brain, and blood samples presented genetic material of *A. reconditum* and that the skin, lung, and liver samples presented a coinfection of *A. reconditum* and* D. immitis* (Fig. 3b,c).

## Discussion

Filariasis is a worldwide parasitic disease affecting both domestic and wild animals as well as humans, and it is necessary to differentiate among etiologic agents for epidemiologic surveillance [12]. Laboratory diagnosis of microfilariae infection has been achieved by detecting circulating parasites, antigens, and/or nucleotides [2].

Microscopic techniques allow the identification of microfilariae in the blood [2]. Knott’s test is the gold standard method [4], and differences in morphology and size of structures allow the discrimination of species. Differential diagnosis is based on the shape of the parasite’s cephalic hook and the posterior part, as well as the length and diameter of the body. However, there have been observed inconsistencies in the filarial organism measurements made using this method [1,2], which may be due to staining and/or fixation of the samples [13].

In this study, the microscopic findings matched those of *D. repens*, *D. immitis*, and *A. reconditum* described by McCall et al. [4] and Otranto et al. [1], and differentiation is difficult, leading to misdiagnosis [14,15]. Therefore, discrimination of microfilariae species requires techniques such as histochemical (e.g., acid phosphatase staining) or molecular methods (e.g., end-point PCR, PCR-ribotyping) [2]. In addition, since the therapy for an *A. reconditum* infection differs from that for heartworm, it is crucial to distinguish between the two [14]. In the present study, *COX1* was used to identify the diversity of filarial species [16], and 5.8s-ITS2-28s rRNA was used for the identification of microfilariae species found in canines, minimizing the requirement for numerous assays and various techniques for species recognition as seen in blood smears. In addition, in samples with low microfilaremia, ITS2 has demonstrated better sensitivity, specificity, and species identification than ITS1 [15]. The coinfection with *D. immitis *and *A. reconditum* found in this patient is consistent with other studies that have reported coinfection of filarial worms [3,17–19], increasing the need for molecular differentiation of these parasites.

In our study, the detection by amplification of the *wsp *gene from the endosymbiont bacterium *Wolbachia *spp. was negative, excluding the presence of this microorganism, like Ionică et al. [3]. The DNA concentration of this bacterium is probably below the detection limits of conventional PCR.

*Acanthocheilonema reconditum *is a species that generally does not require treatment; therefore, its identification prevents inappropriate medical treatment. All heartworm-infected dogs, however, have the potential to infect people, making them a public health concern [1,20]. Therefore, identification of *A. reconditum* in pets may justify treatment to reduce potential transmission to humans [1].

The presence of *Schistosoma haematobium *eggs in the wall of the urinary bladder and *Schistosoma japonicum* eggs in the wall of the large intestine is responsible for squamous cell carcinomas, and *Opisthorchis* and *Clonorchis* can cause cholangiocarcinomas [21]. In the case of filarial organisms, the immune response produced against them is characterized by granulomatous inflammation around the parasite, which can form a nodule [21], which has been evidenced in intra-abdominal surgical procedures or between subcutaneous tissues in adult worms causing nodules formations [12]. In the present case, the patient presented with splenic lymphoma, and the PCR results demonstrated the presence of *D. immitis* and *A. reconditum* in this organ. The presence of these parasites in the neoplastic spleen could be explained by several mechanisms, including their transmigration, lymphatic blockage due to scarring, inflammatory damage, trauma, or stasis, as well as neovascularization that favors parasite concentration in the tumor [9]. However, the coexistence of microfilariae in human patients with neoplasms, including lymphomas, has been reported [9,10–21], considering the presence of the parasite as a predisposing factor or opportunistic pathogen in immunocompromised patients with cancer [10], so it is necessary to carry out studies that demonstrate whether the presence of these filariae is related to the development of tumors and if the coinfection leads to an interaction of these parasites that may increase the possibility of generating neoplasia. Recently, Fercoq et al. [22] demonstrated that in gerbils, the formation of polyps in the pleura is caused by the presence of adult filariae, mainly gravid females, which supports the hypothesis about the role of microfilariae in the development of tumors. Nevertheless, for small domestic animals, no studies are exploring this subject.

In most cases, microfilariae are not found directly in the tumor under the microscope [11]. In our case, even though *A. reconditum* DNA was detected in the spleen, and there was no evidence of the parasite in the cytology or histopathology of the spleen, highlighting the need to implement molecular techniques.

## Conclusion

This study showed for the first time, using molecular methods, a microfilariae mixed infection with *A. reconditum* and *D. immitis* in a dog with a lymphoma. They highlighted the relevance of molecular techniques in diagnosis and the studies about the role of parasites in the development of neoplasia.

## List of Abbreviations

CDV, canine distemper virus; *COX1*, cytochrome oxidase C subunit 1; gDNA, genomic DNA; PCR, polymerase chain reaction; rRNA, ribosomal RNA.

## References

[1] Otranto D, Brianti E, Dantas-Torres F, Weigl S, Latrofa MS, Gaglio G (2011). Morphological and molecular data on the dermal microfilariae of a species of *Cercopithifilaria* from a dog in Sicily. Vet Parasitol.

[2] Magnis J, Lorentz S, Guardone L, Grimm F, Magi M, Naucke TL (2013). Morphometric analyses of canine blood microfilariae isolated by the Knott’s test enables *Dirofilaria immitis* and *D. repens* species-specific and *Acanthocheilonema* (syn. Dipetalonema) genus-specific diagnosis. Parasit Vectors.

[3] Ionică AM, Matei IA, Mircean V, Dumitrache MO, D‘Amico G, Győrke A (2015). Current surveys on the prevalence and distribution of *Dirofilaria* spp. and *Acanthocheilonema reconditum* infections in dogs in Romania. Parasitol Res.

[4] McCall JW, Genchi C, Kramer LH, Guerrero J, Venco L (2008). Heartworm disease in animals and humans. Adv Parasitol.

[5] McCown ME, Monterroso VH, Cardona W (2015). Monitoreo de *Ehrlichia canis Anaplasma phagocytophilum Borrelia burgdorferi*, y *Dirofilaria immitis* en perros de tres ciudades en Colombia. Ces Med Vet Zoo.

[6] Pérez-Ramírez RD, Lugo-Vargas R, Petano-Duque JM, Cruz-Méndez JS, Rondón-Barragán IS (2023). First study on microscopic and molecular detection of *Acanthocheilonema reconditum* and *Leishmania infantum* coinfection in dogs in Southwest Colombia. Vet World.

[7] Vail DM, Thamm DH, Liptak JM (2020). Hematopoietic tumors. Withrow and MacEwen‘s small animal clinical oncology.

[8] Zandvliet M (2016). Canine lymphoma: a review. Vet Quart.

[9] Sahoo N, Saha A, Mishira P (2017). Coexistence of microfilaria with metastatic adenocarcinomatous deposit from breast in axillary lymph node cytology: a rare association. J Cytol.

[10] Agarwal K, Chhikara A (2019). Filarial pleural effusion with lymphoma: a rare association. J Clin Diagn Res.

[11] Chakraborty S, Saha M, Pradhan SG, Biswas S (2019). Microfilaria infection in metastatic node in a case of breast carcinoma. J Mid-Life Health.

[12] Tahir D, Davoust B, Parola P (2019). Vector-borne nematode diseases in pets and humans in the Mediterranean Basin: an update. Vet World.

[13] Liotta JL, Sandhu GK, Rishniw M, Bowman DD (2013). Differentiation of the Microfilariae of *Dirofilaria immitis* and *Dirofilaria repens* in stained blood films. J Parasitol.

[14] Engelmann AM, Schafer AS, Lhamas CL (2019). Morphological and molecular identification of *Acanthocheilonema reconditum* in a canine. Comp Clin Pathol.

[15] Ferreira C, Afonso A, Calado M, Maurício I, Alho AM, Meireles J (2017). Molecular characterization of *Dirofilaria* spp. circulating in Portugal. Parasit Vectors.

[16] Oh IY, Kim KT, Sung HJ (2017). Molecular detection of *Dirofilaria immitis* specific gene from infected dog blood sample using polymerase chain reaction. Iran J Parasitol.

[17] Laidoudi Y, Ringot D, Watier-Grillot S, Davoust B, Mediannikov O (2019). A cardiac and subcutaneous canine dirofilariosis outbreak in a kennel in central France. Parasite.

[18] Ionică AM, Matei IA, D’Amico G, Bel LV, Dumitrache MO, Modrý D (2017). *Dirofilaria immitis* and *D. repens* show circadian co-periodicity in naturally co-infected dogs. Parasit Vectors.

[19] Mircean M, Ionică AM, Mircean V, Györke A, Codea AR, Tăbăran FA (2017). Clinical and pathological effects of *Dirofilaria repens* and *Dirofilaria immitis* in a dog with a natural co-infection. Parasitol Int.

[20] Risch F, Ritter M, Hoerauf A, Hübner MP (2021). Human filariasis-contributions of the *Litomosoides sigmodontis* and *Acanthocheilonema viteae* animal model. Parasitol Res.

[21] Borkowski PK, Rymkiewicz G, Golebiewska J, Nestoros N, Romejko-Jarosinska J, Zarnowska-Prymek H (2015). The first case of human autochtonous subconjunctival dirofilariosis in Poland and MALT lymphoma as possible consequence of this parasitosis. Infect Agents Cancer.

[22] Fercoq F, Remion E, Vallarino-Lhermitte N, Alonso J, Raveendran L, Nixon C (2020). Microfilaria-dependent thoracic pathology associated with eosinophilic and fibrotic polyps in filaria-infected rodents. Parasit Vectors.

